# The –SH Protection Method for Determining Accurate K_*d*_ Values for Enzyme-Coenzyme Complexes of NAD^**+**^-Dependent Glutamate Dehydrogenase and Engineered Mutants: Evidence for Nonproductive NADPH Complexes

**DOI:** 10.4061/2010/951472

**Published:** 2010-06-29

**Authors:** Joanna Griffin, Paul C. Engel

**Affiliations:** School of Biomolecular and Biomedical Science, Conway Institute, University College Dublin, Belfield, Dublin 4, Ireland

## Abstract

Inactivation rates have been measured for clostridial glutamate dehydrogenase and several engineered mutants at various DTNB concentrations. Analysis of rate constants allowed determination of K_d_ for each non-covalent enzyme-DTNB complex and the rate constant for reaction to form the inactive enzyme-thionitrobenzoate adduct. Both parameters are sensitive to the mutations F238S, P262S, the double mutation F238S/P262S, and D263K, all in the coenzyme binding site. Study of the effects of NAD^+^, NADH and NADPH at various concentrations in protecting against inactivation by 200 *μ*M DTNB allowed determination of K_*d*_ values for binding of these coenzymes to each protein, yielding surprising results. The mutations were originally devised to lessen discrimination against the disfavoured coenzyme NADP(H), and activity measurements showed this was achieved. However, the K_*d*_ determinations indicated that, although K_*d*_ values for NAD^+^ and NADH were increased considerably, K_*d*_ for NADPH was increased even more than for NADH, so that discrimination against binding of NADPH was not decreased. This apparent contradiction can only be explained if NADPH has a nonproductive binding mode that is not weakened by the mutations, and a catalytically productive mode that, though strengthened, is masked by the nonproductive binding. Awareness of the latter is important in planning further mutagenesis.

## 1. Introduction

Some years ago we showed that protection of an enzyme against inactivation by Ellman's reagent, DTNB, can be used to obtain very precise measurements of dissociation constants for protecting ligands [1]. The method depends on the most sensitively and accurately measurable property of an enzyme, namely its catalytic activity, and a particularly attractive feature is the fact that it can readily be used under conditions where direct spectrophotometric measurements of ligand binding fail, and with equal ease, regardless of whether the binding is weak or strong. Its application in general requires a chemical modification agent that competes with the ligand for occupation of the same space on the enzyme surface. We show here how the method is applied to a series of mutants affecting the binding of the ligand in question. 

 Glutamate dehydrogenase from *Clostridium symbiosum* [EC 1.4.1.2] (GDH) contains only two cysteine residues per polypeptide chain [2], Cys-144 in helix *α*_7a_ of domain I, the substrate-binding domain, and Cys-320 in a loop that connects *β*_k_ and *α*_13_ in domain II, the coenzyme-binding domain [3]. Cys-144 is remote from the active site, whereas Cys-320, on the surface of domain II, is close to the nicotinamide binding site in the active site cleft and is accessible [4] to 5,5′-dithiobis 2-nitrobenzoic acid (DTNB, Ellman's reagent [5]). DTNB reacts with free sulphydryl groups (–SH) of proteins, forming protein-bound disulphide-linked thionitrobenzoate and releasing 1 mole of thionitrobenzoate (TNB) anion per –SH group. The concentration of the yellow TNB anion can be determined from its absorption at 412 nm, using the molar extinction coefficient of 13.6 mM^−1^cm^−1^ [5]. In chemical modification studies [4], DTNB inactivated clostridial GDH with 1 : 1 stoichiometry on a subunit basis. Site-directed mutagenesis studies [6] confirmed that this inactivation is due to reaction with Cys-320, since the C320S mutant was unaffected by DTNB. Although Cys-320 is not required for catalysis, a bulky substituent at this position prevents coenzyme binding. This explains both why DTNB totally inactivates the enzyme and why NAD^+^ and NADH protect against this inactivation [4].

Basso and Engel [1] showed how this protection can be used to determine coenzyme dissociation constants. Formation of an initial noncovalent enzyme-DTNB complex was reflected in saturation kinetics, that is, increasing DTNB concentration produced a limiting pseudo-first-order rate constant for modification:


(1)
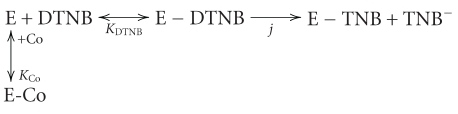

In (1) Co can be NAD(P)H or NAD(P)^+^and it is assumed that, in the absence of a conenzyme, rapid, reversible binding of DTNB is followed by a slower, first-order covalent process governed by a true rate constant *j*. The reacting species is the noncovalent enzyme-DTNB complex, and the apparent first-order rate constant, *k*, for the overall reaction is given by

(2) $$k=\frac{j\left[\text{DTNB}\right]}{K_{\text{DTNB}}+\left[\text{DTNB}\right]}$$

where *K*_DTNB_ is the dissociation constant of the enzyme-DTNB complex. A plot of 1/*k* against 1/[DTNB] gives an ordinate intercept corresponding to 1/*j*, and an abscissa intercept of –1/*K*_DTNB_. 

In the presence of the coenzymes we assume that the enzyme is initially distributed between three species: E, E-Co, and E-DTNB (1). The observed rate constant *K*_Co_ for inactivation by DTNB in the presence of the coenzyme is thus described by

(3) $$\frac{1}{k_{\text{Co}}}=\frac{1}{j}\frac{K_{\text{DTNB}}}{\left[\text{DTNB}\right]}\frac{\left[\text{Co}\right]}{K_{d}}+\frac{1}{k_{\max\;}}$$

predicting that a plot of 1/*k*_Co_ against [Co] should give a straight line with an ordinate intercept of 1/*k*_max_ and slope (1/*j*)(*K*_DTNB_/[DTNB])(1/*K*_*d*_).

Calculation of the dissociation constant for the coenzyme thus requires knowledge of the dissociation constant for DTNB (*K*_DTNB_) and the first-order rate constant for the covalent process (*j*), both obtained from (2). These constants were determined previously for clostridial GDH at pH 7 and 25°C [1], but the present study applies the method to a series of mutants deliberately altered in the vicinity of the coenzyme binding site with a view to altering specificity by improving the binding of NADP(H) [7]. It is likely that values of *K*_DTNB_ and *j* will be different for each variant and accordingly these constants were determined for each of the mutant enzymes.

## 2. Materials and Methods

### 2.1. Materials

The overexpression and dye-affinity purification of clostridial GDH has been described elsewhere [2, 8]. The detailed description of the mutants used in this study is given in [7]. DTNB was from Sigma Chemical Co. (UK). Coenzymes from Roche Biochemicals (98% purity) were used without further purification.

### 2.2. Incubation with DTNB

The enzymes (final concentration 0.1 mg/ml) were incubated in 0.1 M potassium phosphate, pH 7.0 at 25°C, first with DTNB at a range of concentrations from 50 *μ*M up to 600 *μ*M, to determine the baseline parameters *K*_DTNB_ and *j* in (2) for each protein. Each enzyme was then incubated under the same conditions with 200 *μ*M DTNB and various concentrations of coenzymes. Chemical modification reactions were initiated by the addition of DTNB pre-equilibrated to the same temperature and were monitored in two ways. For the lower concentrations of DTNB (25–500 *μ*M), progressive loss of catalytic activity was monitored by standard assay (1 mM NAD^+^, 40 mM L-glutamate, 0.1 M potassium phosphate, and pH 7.0 at 25°C) of 5 *μ*l samples. At higher DTNB concentrations (500–5000 *μ*M), particularly for the mutant enzymes, inactivation was very rapid, and, instead, the release of thionitrobenzoate was continuously monitored at 412 nm in a Cary 50 spectrophotometer with the cell chamber maintained at 25°C. To confirm that both methods measure the same process, both approaches were adopted for two intermediate DTNB concentrations [1]. The stoichiometries of the reactions were calculated from ΔA_412_ values by using the extinction coefficient of 13.6 mM^−1^cm^−1^ [5] for the thionitrobenzoate anion. To determine rate constants, time courses of reactions were fitted by linear regression using the Sigma Plot package (Jandel Scientific GmbH, Erkrath, Germany). Dissociation constants for the coenzymes were estimated according to (3) [1].

## 3. Results

### 3.1. Comparison of DTNB Reaction Parameters for Wild-Type and Mutant Enzymes

**Figure 1:**
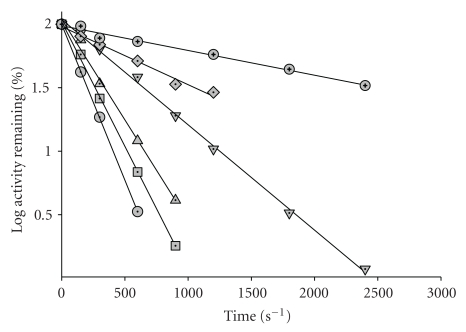
Pseudo-first-order kinetic plots for inactivation of F238S GDH by DTNB.The F2238S GDH mutant (0.1 mg/ml) was incubated in 0.1 M potassium phosphate, pH 7, at 20°C with DTNB at the following concentrations: 500 *μ*M (Lowest line, circles; 400 *μ*M (squares); 300 *μ*M (triangles); 200 *μ*M (inverted triangles); 100 *μ*M (diamonds); 50 *μ*M (upper line, circles).

Typical time courses in Figure 1, in this case for the inactivation of the single mutant F238S by DTNB at different concentrations, illustrate strictly (pseudo) first-order behaviour for inactivation by DTNB. Corresponding results for the wild-type enzyme were in striking agreement with the earlier determination of Basso and Engel [1], a value of 990 (±6.85) *μ*M for *K*_DTNB_ comparing with the earlier figure of 1000 *μ*M. Inactivation of F238S and the double mutant was clearly faster than for the wild-type enzyme. The best fit to the data for F238S gave a limiting value for *j* of 7.95 (±0.22) × 10^−3^ s^−1^ (Table 1), considerably higher than the value of 3.52 (±0.14) × 10^−3^ s^−1^ for the wild-type enzyme. The value for *K*_DTNB_ had also increased by about 20%, indicating that, although this mutant is more accessible to DTNB, this molecule does not bind as tightly to F238S as to the unmutated enzyme. The value of *j* for the P262S mutant was very similar to that for wild-type GDH, but *K*_DTNB_ was approximately 24% lower (Table 1). In the case of the double mutant, the value of *j*, 5.28 (±0.36) × 10^−3^ s^−1^, lies between the figures for the two single mutants. This was also true for the value for *K*_DTNB_, identical within the error to that for wild-type GDH (Table 1). Overall, these results emphasise that, in applying the protection method to determination of ligand dissociation constants with mutant enzymes, it must not be assumed that the baseline values of *j* and *K*_DTNB_ determined for the chemical modification of the unmutated enzyme will also apply to the mutants. Individual values of *j* and *K*_DTNB_ need to be obtained for each protein studied. 

### 3.2. Comparison of Coenzyme Dissociation Constants for Wild-Type and Mutant Enzymes

**Figure 2:**
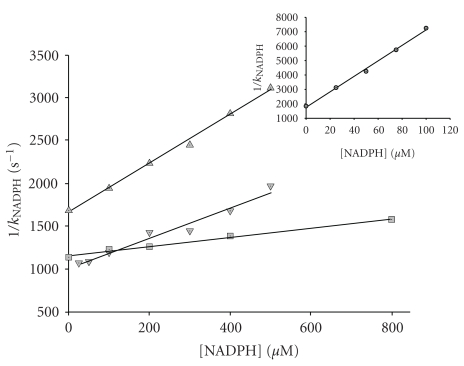
Protection by NADPH against inactivation by DTNB. The plots show for wild-type GDH and three mutants the dependence on NADPH concentration of the pseudo-first-order rate constant *k*_NADPH_ for inactivation of the enzyme by 200 *μ*M DTNB under the same conditions as in Figure 1. The generalised equation giving rise to this plot is (3), *k*_Co_ in this case being *k*_NADPH_. The inset plot shows data for wild-type GDH. In the main plot the data are for F238S (squares), P262S (triangles, upper line), and F2238S/P262S (inverted triangles).

Armed with the values of *K*_DTNB_ and *j* for each protein, one can now proceed to analyse the protection by coenzymes. In each case the protein was incubated with 200 *μ*M DTNB in the presence of different concentrations of each of the coenzymes studied. Each time course yielded a pseudo-first-order inactivation constant that gradually decreased with increasing concentration of the protecting coenzyme. The reciprocals of the observed rate constants were plotted against the reciprocals of the concentrations of the coenzymes, and the plots were analysed according to (3).  Figure 2 shows representative plots, with NADPH as the protecting ligand, for wild-type GDH and the mutants F238S, P262S, and F2328S/262S. In all cases these plots were convincingly linear, allowing extraction of the corresponding coenzyme dissociation constants, which are presented in Table 2. The K*_d_* value thus derived for NAD^+^ in its binary complex with the wild-type enzyme (0.335 mM) is remarkably similar to the earlier estimate of 0.330 mM [1], giving considerable confidence in the reliability and robustness of the method. A more general examination of dissociation constants for NAD^+^, NADH, and NADPH, however, reveals some surprises. The prediction guiding the mutagenesis was that the mutations at positions 238 and 262 should provide more space and a more polar environment in the potential binding pocket for accommodating the additional phosphate in NADP(H), and that the replacement of negative by positive charge in the mutant D263K should help to stabilise the phosphate. In fact, for all three coenzymes tested here, the K*_d_* values for the mutants had increased compared to those for the wild-type enzyme (Table 2). Moreover there was no indication of a decrease in discrimination against binding of NADPH as opposed to NADH. To the contrary, for the F238S mutant there was an approximately 20-fold increase in the K*_d_* for NAD^+^, 10-fold for NADH, and 56-fold for NADPH (Figure 2), respectively. In this instance, where the wild-type enzyme had comparable K*_d_* values for the reduced coenzymes, the F238S mutation resulted in a 5.6-fold increase in the binding discrimination against NADPH (Table 2).

The P262S mutant also displayed an increase in K*_d_* with NAD^+^ but not as large (8.5-fold) as that obtained for the F238S enzyme. P262S also showed increases in the dissociation constants for the reduced coenzymes by factors of 2.1 and 16 for NADH and NADPH, respectively. This repeats the pattern seen with F238S, where the adverse effect on binding of the adenosine moiety of NAD^+^ is less pronounced with the reduced cofactor. The favourable binding of the reduced nicotinamide ring should be unaffected by the two mutations. In the case of the double mutant F238S/P262S, there were again increases in measured dissociation constants for all three coenzymes, 13-fold for NAD^+^, approximately 10-fold for NADH, and 22.5-fold for NADPH. 

Inactivation of the D263K mutant resulted in a substantially decreased value for *K*_DTNB_ of 338 ± 14.5 *μ*M and the value for *j* was 2.5 (± 0.11) × 10^−3^ s^−1^, 30% lower than for wild-type (Table 2). Examination of dissociation constants for the mutant enzyme (Table 2) revealed an increase in K*_d_* for all of the three coenzymes tested, 6-fold for NAD^+^, 5-fold for NADH, and 10-fold increase for NADPH. 

## 4. Discussion

Since Cys-320 is in the coenzyme binding site of clostridial GDH and its modification with DTNB prevents coenzyme binding, we assume conversely that these studies of protection by the coenzyme(s) against inactivation by DTNB report on coenzyme binding at the active site. The enzyme variants examined in this paper were all altered in the adenosine binding portion of the coenzyme binding site as judged from the solved crystal structure for the enzyme-NAD^+^ binary complex [3]. As might be expected for mutations in this location, all of them resulted in substantial changes in the coenzyme dissociation constants. Also in keeping with expectation was the weakening of the binding of NAD^+^ and NADH in every case. However, the results with NADPH were unexpected and entirely contrary to prediction. As mentioned above, the mutations were designed to accommodate the additional phosphate of NADP(H) in the predicted binding pocket, and indeed the kinetic analysis of these mutants [7] shows that the changes achieved their objective of diminishing the discrimination against the phosphorylated cofactor. There is thus an apparent conflict between the functional measurements of activity, which show that the mutations have eased the acceptance of NADPH, and the measurements of binding constants that suggest that they have not. The most obvious explanation of this diametrical opposition lies in the possible falsehood of the underlying assumption that the coenzyme molecule has only one mode of binding at the active site. It is entirely possible and maybe even probable that the “wrong” cofactor, NADPH, without the correct binding pocket to receive its 2′-phosphate, finds an alternative and catalytically unproductive way of occupying the coenzyme site. If so, the introduced mutations could weaken overall binding while selectively strengthening productive binding. If this analysis is correct, it carries an important implication for protein engineering experiments. The unproductive mode of binding would be, in effect, in direct competition with the productive mode, and this means that it is of importance not only to concentrate on strengthening the latter but also to give attention to weakening the former. Further analysis of new mutants and especially of new structures of binary complexes may be required to resolve this puzzle. 

Methodologically, the protection method employed here for determining the dissociation constants has proved its worth. Each K*_d_* determination requires a considerable number of rate measurements, but the resulting constants emerge with excellent precision. The method could be employed in any situation where an active site-directed inhibitor competes directly and cleanly with a substrate for access to its binding site. Ellman's reagent offers particular advantages for a protein with a single modifiable cysteine in the active site. Where there is no reactive cysteine, it may be worth contemplating the mutagenic substitution of a strategically placed Ser by Cys in order to make a protein amenable to this useful method.

## Figures and Tables

**Table 1:** Comparison of limiting values of the first-order rate constants (*j*) for saturating levels of DTNB for wild-type GDH and mutant variants, and of dissociation constants governing binding of DTNB to the enzymes (*K*_DTNB_).

	*j* (s^−1^) × 10^3^	*K*_DTNB_ (*μ*M)
Wild-type GDH	3.52 ± 0.14	990 ± 6.8
F238S	7.95 ± 0.22	1190 ± 17.5
P262S	3.45 ± 0.17	758 ± 23.1
F238S/P262S	5.28 ± 0.36	981 ± 13.4
D263K	2.50 ± 0.12	338 ± 14.5

**Table 2:** Comparison of dissociation constants of wild-type and mutant enzymes for different coenzymes.

	*K*_*d*NAD+_ (mM)	*K*_*d*NADH_ (mM)	*K*_*d*NADPH_ (mM)	*K*_*d*NADPH_/*K*_*d*NADH_
Wild-type	0.335 ± 0.017	0.024 ± 0.001	0.023 ± 0.001	0.96
F238S	6.62 ± 0.25	0.232 ± 0.056	1.30 ± 0.056	5.6
P262S	2.84 ± 0.22	0.052 ± 0.004	0.375 ± 0.03	7.2
F238S/P262S	4.30 ± 0.38	0.253 ± 0.021	0.526 ± 0.045	2.1
D263K	2.02 ± 0.21	0.130 ± 0.012	0.228 ± 0.021	1.75
